# Supplementary material for: Evaluation of Parallel Accumulation-Serial Fragmentation methods for metaproteomics using a model microbiome
Source: bioRxiv. 2026 Sep 30:2025.08.13.670166. Preprint. [Version 2] doi: 10.1101/2025.08.13.670166 (PMC13648379; doi:10.1101/2025.08.13.670166)
Supplement: Supplementary file 1 [file media-1.pdf]

SUPPLEMENTARY MATERIAL:

Evaluation of Parallel Accumulation-Serial Fragmentation methods for metaproteomics using a model microbiome

Ruben Shreshta[1], Andrew T. Rajczewski[2], Aleksey Ogurtsov[3], Katherine Do[2], Gelio Alves[3], Yi-Kuo Yu[3], Matthew Willets[1], Manuel Kleiner[4], Timothy J. Griffin[2] and Pratik D. Jagtap[2].

1. *Bruker Scientific LLC, Billerica, MA*
2. *University of Minnesota, Minneapolis, MN*
3. *National Institutes of Health, Bethesda, MD*
4. *Department of Plant and Microbial Biology, North Carolina State University, Raleigh, NC*

Supplementary Table 1

Protein sequence database components used in the proteomic analyses of composite microbiome samples.

Label	Name and strain	Protein input per biological replicate (µg)	Protein abundance %
Ne1	***Nitrosomonas europaeae* ATCC 19718**	60.58	0.082
F2	**Phage F2**	62.04	0.084
F0	**Phage F0**	65.28	0.088
ES18	**Phage ES18 (H1)**	65.55	0.088
P22	**Phage P22 (HT105)**	78.77	0.106
M13	**Phage M13**	109.02	0.147
Nm1	***Nitrosospira multiformis* ATCC 25196**	155.16	0.209
BXL	***Burkholderia xenovorans* LB400**	321.37	0.433
Nu1	***Nitrosomonas ureae* Nm10**	402.68	0.543
BS	***Bacillus subtilis* 168**	583.83	0.788
Nv	***Nitrososphaera viennensis***	607.41	0.819
PaD	***Paracoccus denitrificans* ATCC 17741, (Beijerinck and Minkman) Davis emends. Rainey et al.**	683.66	0.922
DVH	***Desulfovibrio vulgaris* Hildenborough**	701.58	0.946

**Am2**	***Alteromonas macleodii* ATCC 27126**	707.47	0.954
**KF7**	***Pseudomonas pseudoalcaligenes* KF707**	863.8	1.165
**CV**	***Chromobacterium violaceum* CV026**	933.08	1.259
**199**	***Roseobacter* sp. AK199**	1183.43	1.596
**HB2**	***Thermus thermophilus* HB27**	1245.63	1.68
**259 + 137**	***Staphylococcus aureus* subsp. *aureus* Rosenbach, Seattle 1945, ATCC 25923; *Staphylococcus aureus subsp. aureus Rosenbach, Smith [NCTC 10399], ATCC 13709***	1931.46	2.606
**PD**	***Pseudomonas denitrificans* ATCC 13867**	2128.51	2.871
**VF + 841**	***Rhizobium leguminosarum* bv. *viciae* VF39*; Rhizobium leguminosarum bv. viciae 3841***	2351.5	3.172
**CRH**	***Chlamydomonas reinhardtii***	2962.73	3.996
**ATN**	***Agrobacterium tumefaciens* NTL4**	4186.28	5.647
**K12+ 2191**	***Escherichia coli K12 with Flac+ Plasmid***	4290.54	5.788
**Pfl**	***Pseudomonas fluorescens* ATCC 13525, Type strain**	4964.22	6.696
**SMS**	***Stenotrophomonas maltophilia* SeITE02**	5946.27	8.021
**Cup**	***Cupriavidus metallireducens* CH34; (DSM 2839; LMG 1195; CIP 107179)**	11504.98	15.519
**LT2**	***Salmonella enterica typhimurium* LT2**	25037.78	33.774

Supplementary Table 2

The diaPASEF method covered the range of 300 to 1200m/z and an ion mobility range of 0.7-1.33 Vs cm−2, with 35Da isolation windows (1Da overlap) with a total cycle time of 850 ms. The collision energy (CE) was a linear function between the CE and the ion-mobility starting from 20 eV at 1/K0 = 0.6 Vs cm−2 to 59 eV at 1/K0 = 1.6 V cm−2. Accumulation and ramp time were set to 100 ms.

#MS Type	Cycle Id	Start IM [1/K0]	End IM [1/K0]	Start Mass [m/z]	End Mass [m/z]	CE [eV]
**MS1**	0	-	-	-	-	-
**PASEF**	1	0.7	0.865	404.5	440.5	-

**PASEF**	1	0.865	1.055	649.5	685.5	-
**PASEF**	1	1.055	1.215	894.5	930.5	-
**PASEF**	1	1.215	1.3	1139.5	1200.5	-
**PASEF**	2	0.7	0.836	369.5	405.5	-
**PASEF**	2	0.836	1.029	614.5	650.5	-
**PASEF**	2	1.029	1.189	859.5	895.5	-
**PASEF**	2	1.189	1.3	1099.5	1140.5	-
**PASEF**	3	0.7	0.808	334.5	370.5	-
**PASEF**	3	0.808	1.005	579.5	615.5	-
**PASEF**	3	1.005	1.17	824.5	860.5	-
**PASEF**	3	1.17	1.3	1069.5	1100.5	-
**PASEF**	4	0.7	0.782	299.5	335.5	-
**PASEF**	4	0.782	0.964	544.5	580.5	-
**PASEF**	4	0.964	1.148	789.5	825.5	-
**PASEF**	4	1.148	1.3	1039.5	1070.5	-
**PASEF**	5	0.7	0.938	509.5	545.5	-
**PASEF**	5	0.938	1.126	754.5	790.5	-
**PASEF**	5	1.126	1.3	999.5	1040.5	-
**PASEF**	6	0.7	0.913	474.5	510.5	-
**PASEF**	6	0.913	1.101	719.5	755.5	-
**PASEF**	6	1.101	1.3	964.5	1000.5	-
**PASEF**	7	0.7	0.889	439.5	475.5	-
**PASEF**	7	0.889	1.078	684.5	720.5	-
**PASEF**	7	1.078	1.3	929.5	965.5	-

Supplementary Table 3

Strain-associated variant (SAV) peptides detected by diaPASEF. In cases where both the reference and the associated single amino acid variant (SAAV) were detected, peptide sequences identified experimentally are shown, with the amino acid variant shown in [ ]. Peptides originate from four bacterial strains analyzed in this study: *Staphylococcus aureus* ATCC 13709 (“137”), *S. aureus* ATCC 25923 (“259”), *Rhizobium leguminosarum* bv. *viciae* 3841 (“841”), and *R. leguminosarum* bv. *viciae* VF39 (“VF”). For peptides detected only in a single strain, please refer to the submission in the MassIVE repository.

Peptide	Peptide SAV
**Identified in both *Rhizobium leguminosarum* bv. *viciae* 3481(“841”) and *Rhizobium leguminosarum* bv. *viciae VF39* (“VF”) strains**	
AEGSGDEA[A]MPIR	AEGSGDEA[E]MPIR
AGECHLIPYPNAADV[A]ELK	AGECHLIPYPNAADV[S]ELK
AGLFESV[D]VK	AGLFESV[E]VK
AISDLQAAGQNVSEQDV[A]AK	AISDLQAAGQNVSEQDV[S]AK
AL[N]AAIDAFLK	AL[D]AAIDAFLK
AQDATNANLS[A]ADIDALDK	AQDATNANLS[E]ADIDALDK
ASDVR[G]FTADQLK	ASDVR[A]FTADQLK
AVTPDA[D]HKYDNDFGNEWLK	AVTPDA[E]HKYDNDFGNEWLK
DAKGDPILIDSSFT[A]DPAVITR	DAKGDPILIDSSFT[P]DPAVITR
EAQPEAEQAQPEAKP[D]GGK	EAQPEAEQAQPEAKP[E]GGK
EGD[V]EISGPR	EGD[I]EISGPR
[L]HQNEASPVQK	[F]HQNEASPVQK
GDPILIDSSFT[A]DPAVITR	GDPILIDSSFT[P]DPAVITR
GGINE[A]AEAAADSQSMR	GGINE[V]AEAAADSQSMR
IFVTEF[A]DLMPK	IFVTEF[S]DLMPK
ILGYGL[D]EFR	ILGYGL[E]EFR
IT[D]ISETGGTFDK	IT[E]ISETGGTFDK
IVAEQENADYSG[V]IIR	IVAEQENADYSG[I]IIR
IVAEQENADYSG[V]IIRK	IVAEQENADYSG[I]IIRK
IVFEPNP[D]GDEFK	IVFEPNP[E]GDEFK
IVFEPNP[D]GDEFKFESK	IVFEPNP[E]GDEFKFESK
LLQAL[A]IESSASTFSTLK	LLQAL[S]IESSASTFSTLK
LSLPIWASSYEDPAV[A]K	LSLPIWASSYEDPAV[T]K
MLDPMVVGEEHY[D]VAR	MLDPMVVGEEHY[E]VAR
MLDPMVVGEEHY[D]VARK	MLDPMVVGEEHY[E]VARK
NIPIELAAPTQVAIG[G]GAASLNGLTLK	NIPIELAAPTQVAIG[D]GAASLNGLTLK
NLYDPSPNGYSTAVI[V]PR	NLYDPSPNGYSTAVI[M]PR
QIGELI[D]AYK	QIGELI[E]AYK

SDLAGI[V]ASNK	SDLAGI[M]ASNK
SEADLIE[G]WSTAR	SEADLIE[A]WSTAR
SIDAVYLPEMAFDL[D]AEAAR	SIDAVYLPEMAFDL[E]AEAAR
TGYAGSG[A]YK	TGYAGSG[P]YK
TVED[S]GLSGADYAPHGGSFPINVK	TVED[N]GLSGADYAPHGGSFPINVK
[A]SPEELER	[V]SPEELER
VTRV[D]VTDYQEF	VTRV[E]VTDYQEF
WPA[A]DVESLIR	WPA[E]DVESLIR
AEVA[G]VEAPTAEAR	AEVA[A]VEAPTAEAR
AIGYSDADLDALPQSV[Q]DGK	AIGYSDADLDALPQSV[E]DGK
ALASVPITLDVK[V]V	ALASVPITLDVK[I]V
AMNDVL[A]ALK	AMNDVL[T]ALK
ASMEYLTAR[V]	ASMEYLTAR[I]
AVVAPWDVL[V]FK	AVVAPWDVL[T]FK
[N]FNAAQTGAK	[D]FNAAQTGAK
DYV[A]AAKPAFEK	DYV[T]AAKPAFEK
EALHPYG[G]K	EALHPYG[S]K
EAQPAAP[D]AVTPTAESAKPEATPEAK	EAQPAAP[E]AVTPTAESAKPEATPEAK
EAQPAAP[D]AVTPTAESAKPEATPEAKPAAEAPAEK	EAQPAAP[E]AVTPTAESAKPEATPEAKPAAEAPAEK
ELAAQGYPT[A]GFR	ELAAQGYPT[V]GFR
GLNVGQSD[V]TSDYWQGDEGK	GLNVGQSD[I]TSDYWQGDEGK
GLNVGQSD[V]TSDYWQGDEGKFQK	GLNVGQSD[I]TSDYWQGDEGKFQK
IAATGTIDGQPIG[I]NGDIR	IAATGTIDGQPIG[M]NGDIR
IAVLLQDGESASDMSASAPAAAPAAAPQAAQEEKP[A]AATPASAPVPAEPK	IAVLLQDGESASDMSASAPAAAPAAAPQAAQEEKP[V]AATPASAPVPAEPK
IEPEVSAPPV[K]	IEPEVSAPPV[R]
I[V]EITPLTKPADIIAEIPR	I[L]EITPLTKPADIIAEIPR
LA[A]ALLDLAK	LA[S]ALLDLAK
LEVLT[G]NVK	LEVLT[S]NVK
LGLSITDDPLI[V]R	LGLSITDDPLI[L]R
LIEN[A]AAAGIPAVK	LIEN[T]AAAGIPAVK
LLTDGTEVDSGPATVPGT[P]TAEGK	LLTDGTEVDSGPATVPGT[L]TAEGK
REFIQ[D]NALSVANLDI	REFIQ[E]NALSVANLDI
[A]KQEPLTVFHSALENIAPHVEVR	[S]KQEPLTVFHSALENIAPHVEVR
SNHLLVDANLLQN[K]	SNHLLVDANLLQN[R]
[A]SFEDLGHDLGYALLLK	[S]SFEDLGHDLGYALLLK
SVAENIVYGAFDVV[Q]GK	SVAENIVYGAFDVV[H]GK
SVV[N]TVGGTPLK	SVV[D]TVGGTPLK
[A]YNWVFNKPEA	[T]YNWVFNKPEA
VAQETAPEADLVA[K]	VAQETAPEADLVA[R]
V[V]DPTGQPPGTIVVDTPSR	V[L]DPTGQPPGTIVVDTPSR
VLGVLEGIQ[K]	VLGVLEGIQ[R]

Identified in both *Staphylococcus aureus* ATCC 13709 ("137") and *Staphylococcus aureus* ATCC 25923 ("259") strains	
AEDN[P]FFPSPYSLSQYTAPK	AEDN[A]FFPSPYSLSQYTAPK
AFNEQPFA[I]VK	AFNEQPFA[V]VK
ALNSTIANV[D]EDIEK	ALNSTIANV[N]EDIEK
[V]NLGNQNIMAVAWYQNSAEAK	[A]NLGNQNIMAVAWYQNSAEAK
ATDAEN[L]EK	ATDAEN[V]EK
ATDAEN[L]EKEEAITK	ATDAEN[V]EKEEAITK
AVAGLSE[E]QLK	AVAGLSE[D]QLK
AYKAQGL[S]AWGF	AYKAQGL[G]AWGF
DGEVALFRP[E]ENFK	DGEVALFRP[D]ENFK
DIKDF[D]DVK	DIKDF[N]DVK
DVVVTFPEEYH[T]EELAGK	DVVVTFPEEYH[A]EELAGK
DVVVTFPEEYH[T]EELAGKEATFK	DVVVTFPEEYH[A]EELAGKEATFK
EEASANNLSDTSQEAQ[E]IQEAKK	EEASANNLSDTSQEAQ[A]IQEAKK
EFAQLNGEEIN[E]QPSNFK	EFAQLNGEEIN[D]QPSNFK
EIELEDPYENMG[T]K	EIELEDPYENMG[A]K
EIP[Q]LVTK	EIP[L]LVTK
EIVSEGTDFPDFDAAL[T]AYK	EIVSEGTDFPDFDAAL[A]AYK
EQV[D]QITAIFK	EQV[N]QITAIFK
EVDIDISNHTS[E]LIDNDILK	EVDIDISNHTS[D]LIDNDILK
FTELLV[E]K	FTELLV[D]K
GITTFDLVQ[D]K	GITTFDLVQ[N]K
GTFIIDPDGVVQASEINADGIGRDASTL[S]HK	GTFIIDPDGVVQASEINADGIGRDASTL[A]HK
GY[T]AEQMVEELSQDFTNISK	GY[S]AEQMVEELSQDFTNISK
IAITFSEE[E]NVDRIVQAK	IAITFSEE[G]NVDRIVQAK
IL[D]PNGYWNSTLR	IL[N]PNGYWNSTLR
IVIDFG[I]DK	IVIDFG[V]DK
KAVAGLSE[E]QLK	KAVAGLSE[D]QLK
LHLNDLHDSEISLISTSGTFSD[R]	LHLNDLHDSEISLISTSGTFSD[K]
LLE[S]AGLIK	LLE[A]AGLIK
LSVLG[T]DLLK	LSVLG[A]DLLK
MIAPVLEELA[T]DYEGK	MIAPVLEELA[A]DYEGK
NEGDVT[N]SNYNQNSAVK	NEGDVT[I]SNYNQNSAVK
NGEVSNIKPSLIN[I]K	NGEVSNIKPSLIN[V]K
[D]HLHLVFE	[N]HLHLVFE
NNKPQ[N]QPAAPK	NNKPQ[S]QPAAPK
NNMQEISSELQQSEQ[F]K	NNMQEISSELQQSEQ[S]K
NVE[Y]FTEMPVR	NVE[H]FTEMPVR
NVLSTLEQ[L]K	NVLSTLEQ[P]K
QSALQHNVEVN[D]QDELK	QSALQHNVEVN[N]QDELK
Q[N]FNDILNYGVQIK	Q[S]FNDILNYGVQIK

[T]ISYNQQNYDTIASGK	[S]ISYNQQNYDTIASGK
SNNGLSM[I]PWGTK	SNNGLSM[V]PWGTK
[T]VMYLLVNEGELSTFGPK	[S]VMYLLVNEGELSTFGPK
TAFDPNQSGN[I]FMAANFK	TAFDPNQSGN[T]FMAANFK
TEYHNLTF[T]PAQFETEIVQAK	TEYHNLTF[A]PAQFETEIVQAK
TGGLGASYSTSSNN[I]QVTTTMAPSSNGR	TGGLGASYSTSSNN[V]QVTTTMAPSSNGR
TNTYYVEATNN[N]PK	TNTYYVEATNN[S]PK
TTEVVGE[D]HVTGIR	TTEVVGE[N]HVTGIR
TVILPIV[E]R	TVILPIV[G]R
TYTFVFTDYVN[E]K	TYTFVFTDYVN[D]K
VEAH[L]INNMGHDHTR	VEAH[S]INNMGHDHTR
V[E]LPNGVELR	V[V]LPNGVELR
VYVPND[E]EGR	VYVPND[D]EGR
ADAQQN[K]FNK	ADAQQN[N]FNK
AE[S]QANQMVGDAVEK	AE[A]QANQMVGDAVEK
AFIDSQ[E]FK	AFIDSQ[G]FK
AFIETYQQQHP[E]DEVK	AFIETYQQQHP[D]DEVK
AHETAQN[T]DLK	AHETAQN[A]DLK
AQDSL[N]DNTTK	AQDSL[I]DNTTK
[V]QENGLTVVDAFNFEAPK	[A]QENGLTVVDAFNFEAPK
[T]SQDEVGSGVVYKK	[A]SQDEVGSGVVYKK
[S]VHLTEQGADK	[A]VHLTEQGADK
D[V]DFFNQAK	D[A]DFFNQAK
DAF[E]LPYTIIK	DAF[A]LPYTIIK
DA[T]IDMLGTASKVEVTK	DA[S]IDMLGTASKVEVTK
D[E]NAHPLTQGTFIK	D[G]NAHPLTQGTFIK
DITTIMDNGEAYGYAT[D]K	DITTIMDNGEAYGYAT[N]K
[E]NSEHFNVEIAK	[D]NSEHFNVEIAK
DQQSAFYEILNMPNLNE[E]QR	DQQSAFYEILNMPNLNE[A]QR
DVMT[Y]GGLK	DVMT[F]GGLK
EAGIYE[L]LLTVNK	EAGIYE[S]LLTVNK
EEASANNLSDTSQEAQEIQEAK[R]	EEASANNLSDTSQEAQEIQEAK[K]
EFIQQ[V]PSSPSYALFK	EFIQQ[A]PSSPSYALFK
EKGTEITS[I]FYPK	EKGTEITS[T]FYPK
ELG[T]RTFDEE	ELG[A]RTFDEE
FN[N]AIEASK	FN[S]AIEASK
FN[N]AIEASKVELTK	FN[S]AIEASKVELTK
FP[I]AVIEEVLK	FP[V]AVIEEVLK
GQFHEND[D]IEGVVVR	GQFHEND[V]IEGVVVR
GTEITS[I]FYPK	GTEITS[T]FYPK
HINSGELDGLE[N]EAAITK	HINSGELDGLE[S]EAAITK

IENQEGIDNI[E]EILEVSDGLMVAR	IENQEGIDNI[A]EILEVSDGLMVAR
IGDGD[I]EK	IGDGD[V]EK
IKVTT[L]DELTPLIGK	IKVTT[I]DELTPLIGK
IVNQQS[T]MPAITVALEK	IVNQQS[S]MPAITVALEK
KLDSSITIAD[Y]VVSPTLIHK	KLDSSITIAD[H]VVSPTLIHK
[E]LTQEQIEEAK	[K]LTQEQIEEAK
LGYDTGESETPITPVIIG[E]EK	LGYDTGESETPITPVIIG[D]EK
LNDSQAPKADAQQN[K]FNK	LNDSQAPKADAQQN[N]FNK
LN[E]VEQTNTPGSLNPK	LN[D]VEQTNTPGSLNPK
LTDELVN[V]FQK	LTDELVN[G]FQK
LTFSD[E]VVEK	LTFSD[D]VVEK
MAPQTAGEYEGT[H]YQFK	MAPQTAGEYEGT[N]YQFK
MFA[T]NVPQTTTHK	MFA[A]NVPQTTTHK
NKLTFSD[E]VVEK	NKLTFSD[D]VVEK
NQ[Q]ISYK	NQ[N]ISYK
PYDPN[H]PDNK	PYDPN[N]PDNK
QINPLDNEGLLAFGS[D]LQK	QINPLDNEGLLAFGS[G]LQK
SGEESEVLV[D]DK	SGEESEVLV[A]DK
SGEESEVLV[D]DKNK	SGEESEVLV[A]DKNK
SGTYDANINIADEMF[D]NK	SGTYDANINIADEMF[N]NK
SSA[E]VQQTQQASIPASQK	SSA[D]VQQTQQASIPASQK
TINGTLDLHDELE[E]TLAK	TINGTLDLHDELE[Q]TLAK
TP[T]DSGLEEYAEILSSK	TP[S]DSGLEEYAEILSSK
TTNDAN[N]IATNSELK	TTNDAN[S]IATNSELK
VAVVGQSGNLT[P]ADK	VAVVGQSGNLT[S]ADK
VDRGQQS[D]EDDLNAMK	VDRGQQS[G]EDDLNAMK
VDSTFSYS[L]GGK	VDSTFSYS[S]GGK
VDSTFSYS[L]GGKFDSTK	VDSTFSYS[S]GGKFDSTK
[I]FEQLSSK	[V]FEQLSSK
VVVIPTDEESMIARDVMT[Y]GGLK	VVVIPTDEESMIARDVMT[F]GGLK
YDQTFKGV[T]AK	YDQTFKGV[S]AK
YGINFNQ[T]LETGGVMLGK	YGINFNQ[A]LETGGVMLGK
YSD[Q]NKPYKEG	YSD[D]NKPYKEG
YVFSFKDPN[V]NGK	YVFSFKDPN[A]NGK
YVGD[V]AFLQIEPVEGELNYNK	YVGD[A]AFLQIEPVEGELNYNK

Supplementary Table 4

Strain-associated variant (SAV) peptides detected by ddaPASEF. In cases where both the reference and the associated single amino acid variant (SAAV) were detected, peptide sequences identified experimentally are shown, with the amino acid variant shown in [ ]. Peptides originate from four bacterial strains analyzed in this study: *Staphylococcus aureus* ATCC 13709 (“137”), *S. aureus* ATCC 25923

("259"), *Rhizobium leguminosarum* bv. *viciae* 3841 ("841"), and *R. leguminosarum* bv. *viciae* VF39 ("VF"). For peptides detected only in a single strain, please refer to the submission in the MassIVE repository.

Peptide	Peptide SAV
**Identified in both *Rhizobium leguminosarum* bv. *viciae* 3481("841") and *Rhizobium leguminosarum* bv. *viciae VF39* ("VF") strains**	
AEVA[G]VEAPTAEAR	AEVA[A]VEAPTAEAR
AMNDVL[A]ALK	AMNDVL[T]ALK
EAQPAAP[D]AVTPTAESAKPEATPEAKPAAEAPAEK	EAQPAAP[E]AVTPTAESAKPEATPEAKPAAEAPAEK
GGPFLAELDTFAGVAPDDPAVADL[K]	GGPFLAELDTFAGVAPDDPAVADL[R]
GLNVGQSD[V]TSDYWQGDEGK	GLNVGQSD[I]TSDYWQGDEGK
GLNVGQSD[V]TSDYWQGDEGKFQK	GLNVGQSD[I]TSDYWQGDEGKFQK
IAVLLQDGESASDMSASAPAAAPAAAPQAAQEEKP[A]AATPASAPVPAEPK	IAVLLQDGESASDMSASAPAAAPAAAPQAAQEEKP[V]AATPASAPVPAEPK
I[V]EITPLTKPADIIAEIPR	I[L]EITPLTKPADIIAEIPR
LAYISGQGGQD[G]TGALSPDFAVQVK	LAYISGQGGQD[S]TGALSPDFAVQVK
LLTDGTEVDSGPATVPGT[P]TAEGK	LLTDGTEVDSGPATVPGT[L]TAEGK
LMSV[D]DAIAEGAMALFGEK	LMSV[E]DAIAEGAMALFGEK
REFIQ[D]NALSVANLDI	REFIQ[E]NALSVANLDI
SVAENIVYGAFDVV[Q]GK	SVAENIVYGAFDVV[H]GK
VAQETAPEADLVA[K]	VAQETAPEADLVA[R]
VLGVLEGIQ[K]	VLGVLEGIQ[R]
AISDLQAAGQNVSEQDV[A]AK	AISDLQAAGQNVSEQDV[S]AK
AQDATNANLS[A]ADIDALDK	AQDATNANLS[E]ADIDALDK
A[S]TVGEINEAVMAAANGK	A[T]TVGEINEAVMAAANGK
DAKGDPILIDSSFT[A]DPAVITR	DAKGDPILIDSSFT[P]DPAVITR
GDPILIDSSFT[A]DPAVITR	GDPILIDSSFT[P]DPAVITR
[G]FTADQLKDELAK	[A]FTADQLKDELAK
GGINE[A]AEAAADSQSMR	GGINE[V]AEAAADSQSMR
IVFEPNP[D]GDEFKFESK	IVFEPNP[E]GDEFKFESK
MLDPMVVGEEHY[D]VAR	MLDPMVVGEEHY[E]VAR
MLDPMVVGEEHY[D]VARK	MLDPMVVGEEHY[E]VARK
NLYDPSPNGYSTAVI[V]PR	NLYDPSPNGYSTAVI[M]PR
RGAEGEEAAAEGEGAS[A]EAPVLADVASE	RGAEGEEAAAEGEGAS[T]EAPVLADVASE
SDLAGI[V]ASNK	SDLAGI[M]ASNK
SEADLIE[G]WSTAR	SEADLIE[A]WSTAR
TLDELNAYAH[A]K	TLDELNAYAH[T]K
**Identified in both *Staphylococcus aureus* ATCC 13709 ("137") and *Staphylococcus aureus* ATCC 25923 ("259") strains**	
EDSKE[E]QIK	EDSKE[A]QIK
EDSKE[E]QIKK	EDSKE[A]QIKK
E[I]QIKKSFAK	E[G]QIKKSFAK

EDSKE[I]QIK	EDSKE[A]QIK
EDSKE[I]QIKK	EDSKE[A]QIKK
EDSKE[E]QIK	EDSKE[G]QIK
EDSKE[I]QIK	EDSKE[G]QIK
EDSKE[E]QIKK	EDSKE[G]QIKK
EDSKE[I]QIKK	EDSKE[G]QIKK
EKQK[T]IYYK	EKQK[A]IYYK
IGD[F]FSK	IGD[L]FSK
MK[F]TAIAK	MK[L]TAIAK
MNKN[L]VIK	MNKN[I]VIK
QK[T]IYYK	QK[A]IYYK
SVLKS[E]R	SVLKS[D]R
SVLKS[E]R	SVLKS[N]R

Supplementary Figure 1

A

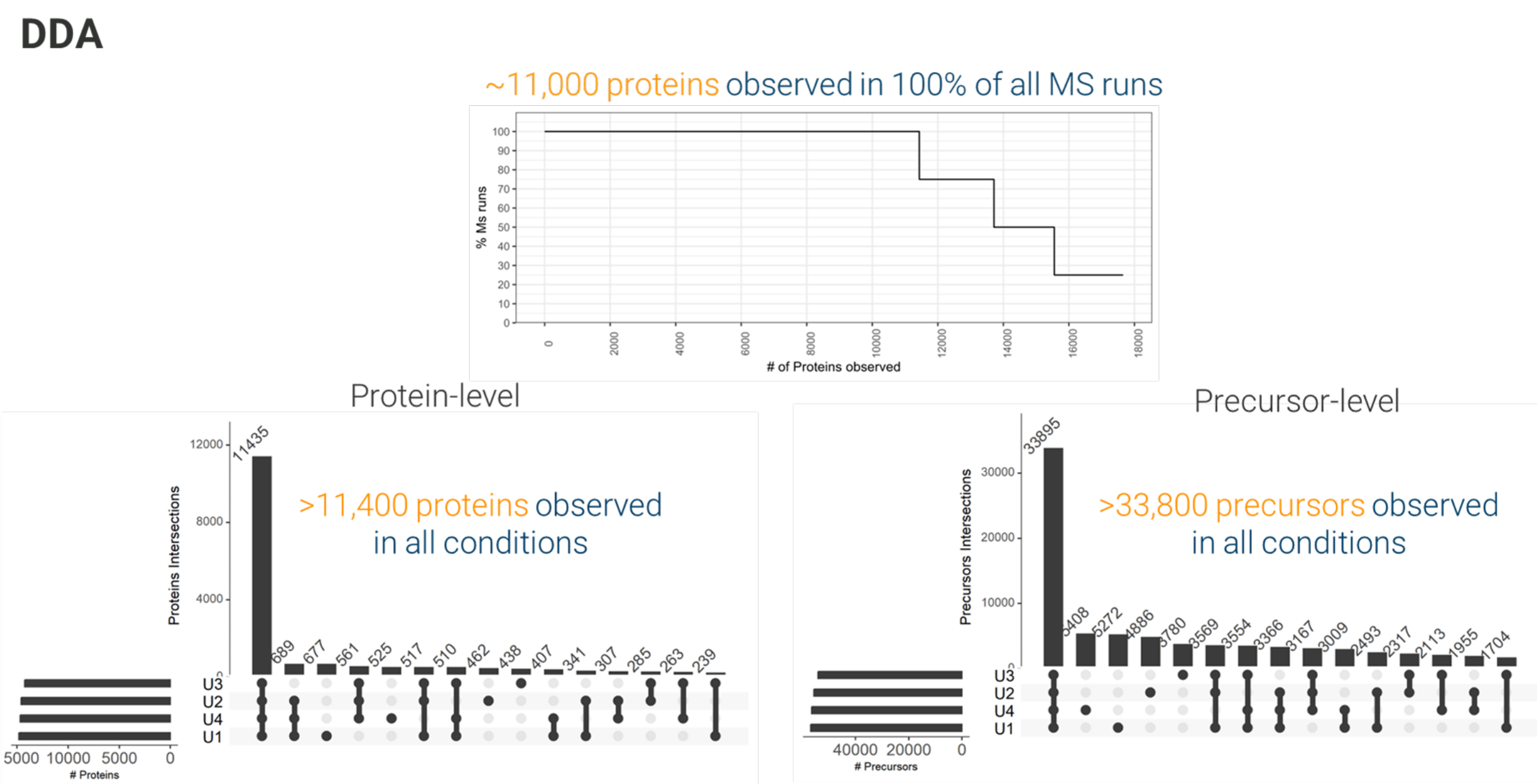

B

DIA

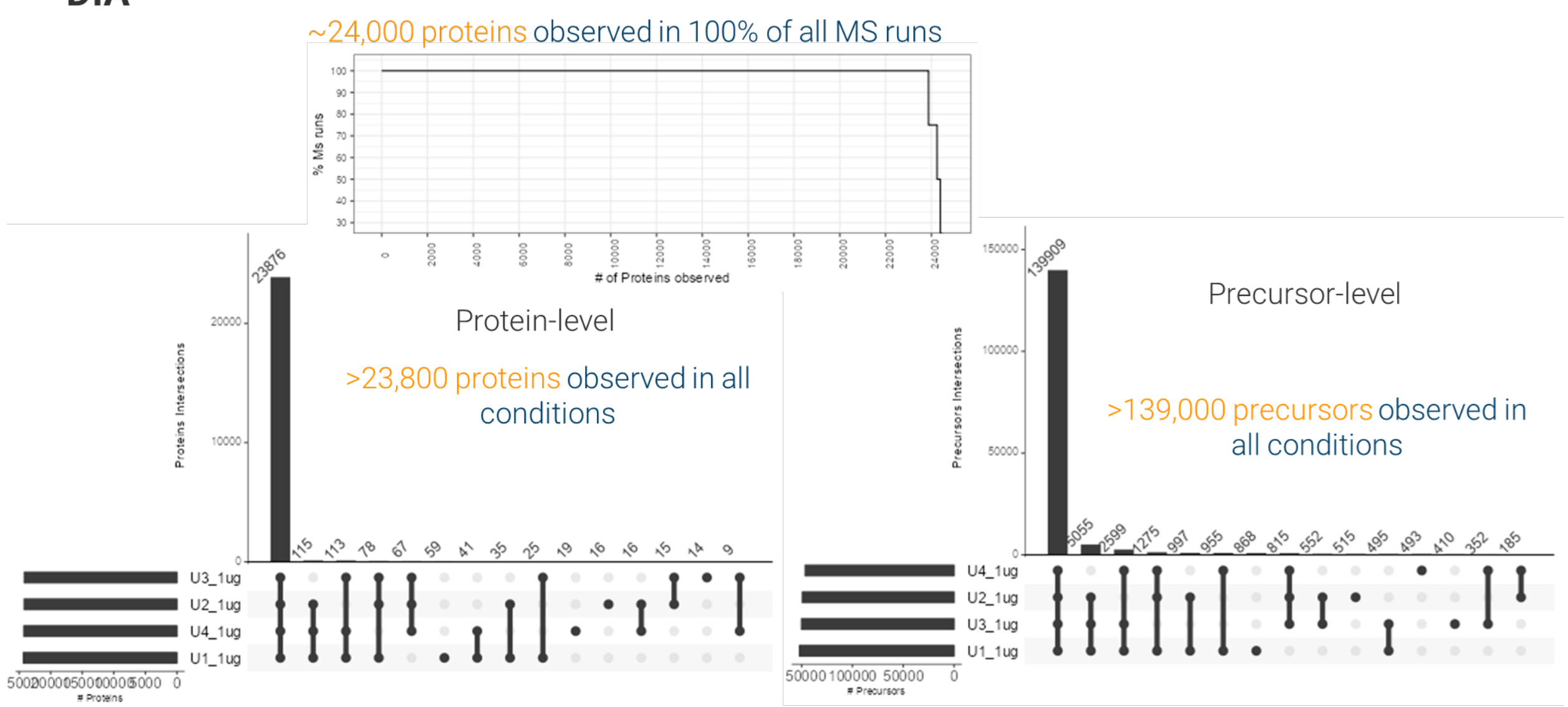
~24,000 proteins observed in 100% of all MS runs
% MS runs
# of Proteins observed
Protein-level
>23,800 proteins observed in all conditions
Proteins Intersections
23876
115
113
78
67
59
41
35
25
19
16
16
15
14
9
U3_1ug
U2_1ug
U4_1ug
U1_1ug
Proteins
Precursor-level
>139,000 precursors observed in all conditions
Precursors Intersections
139909
5055
2599
1275
997
955
868
815
552
515
495
493
410
352
185
U4_1ug
U2_1ug
U3_1ug
U1_1ug
Precursors

C

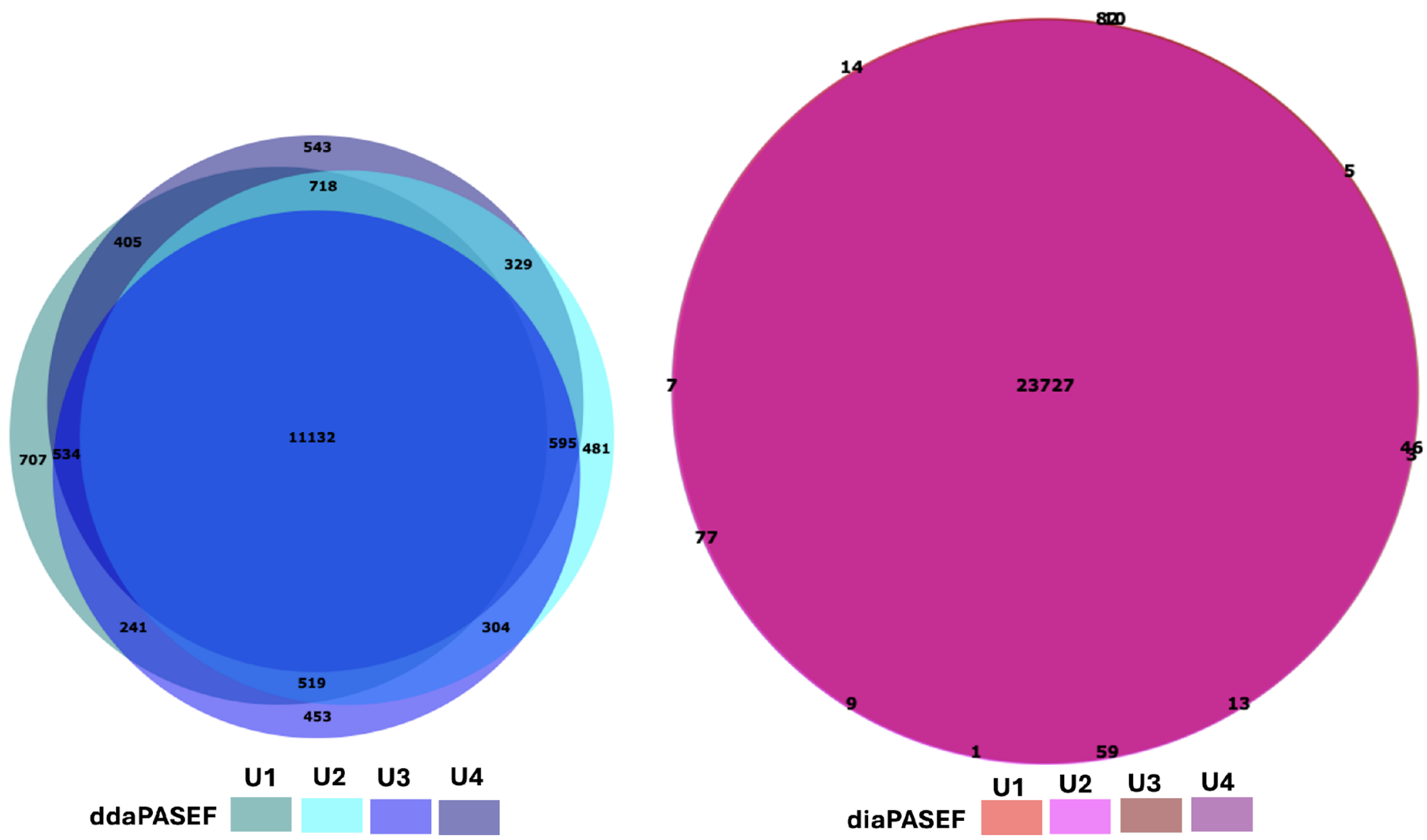
543
718
405
329
707
534
11132
595
481
241
304
519
453
U1
U2
U3
U4
ddaPASEF
14
5
7
23727
77
9
13
1
59
U1
U2
U3
U4
diaPASEF

Supplementary Figure 1. Comparison of precursors and proteins observed using A) ddaPASEF (DDA) and B) diaPASEF (DIA) methods across four replicates of uneven mock community samples (U1–U4). C) Venn diagram of proteins with quantitative values in ddaPASEF data and diaPASEF data.

Supplementary Figure 2

A

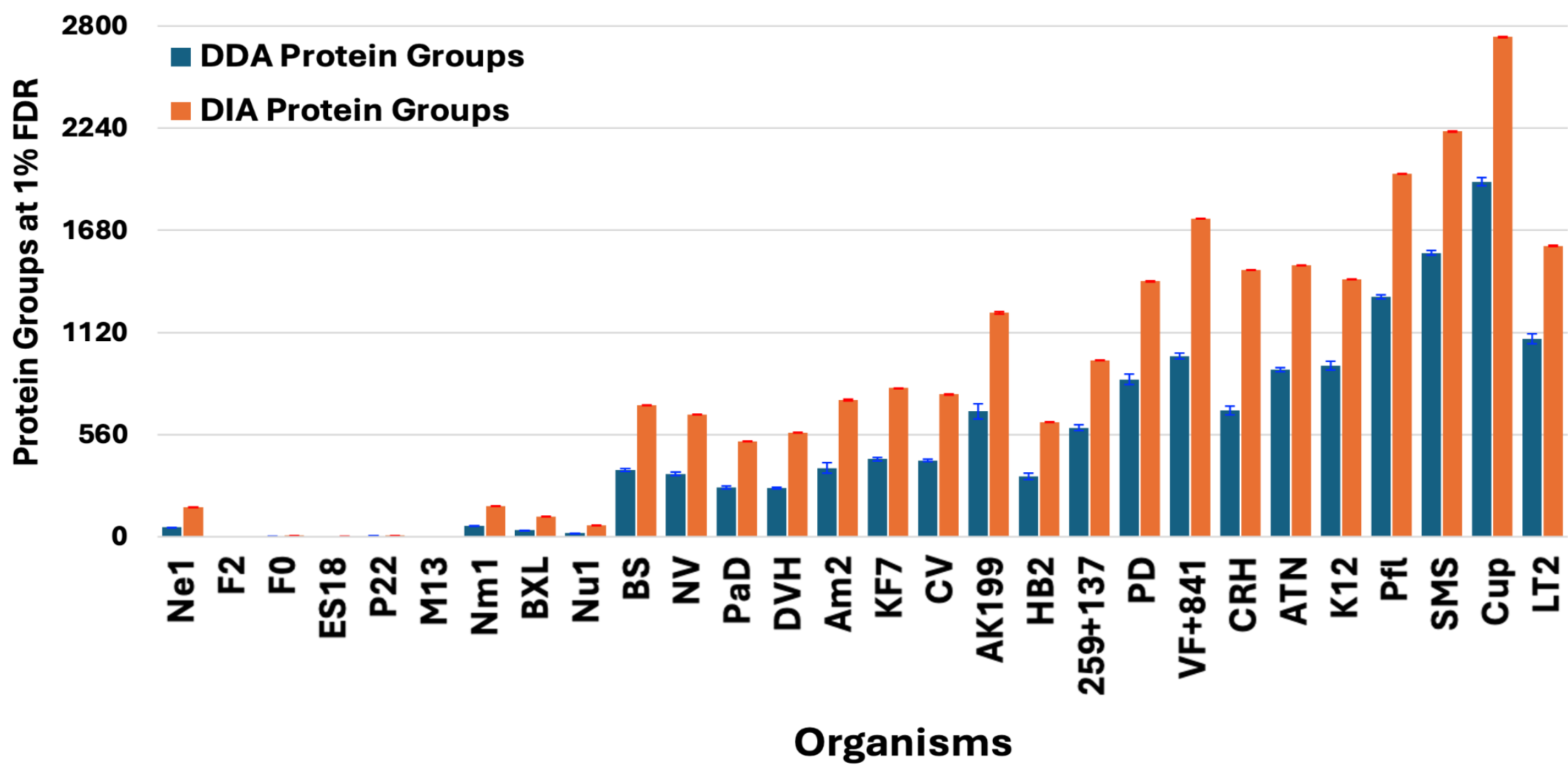

B

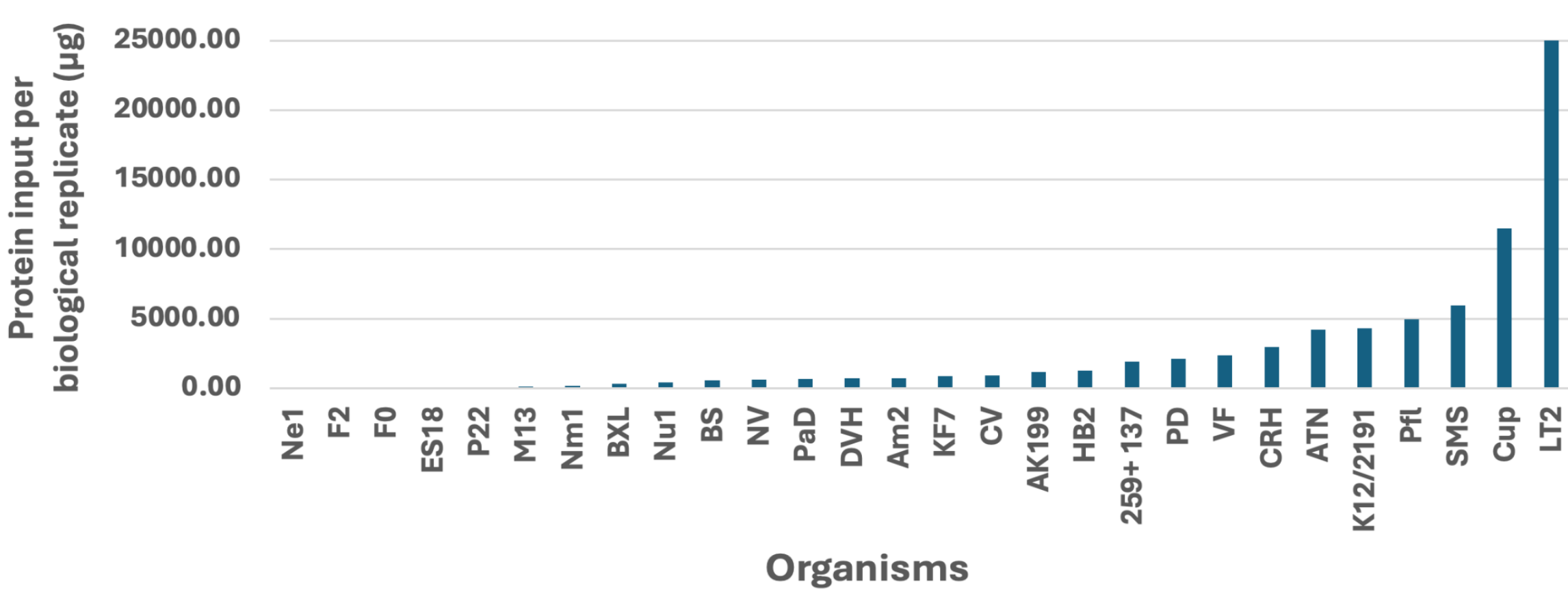

Supplementary Figure 2: A) Comparison of ddaPASEF and diaPASEF protein groups by organism. Average protein groups were obtained from ddaPASEF (DDA, blue) and diaPASEF (DIA, orange) searches using SpectroMine and Spectronaut, respectively. Organisms were ordered along the x-axis by protein abundance (smallest to largest). **B) Comparison of protein input per biological replicate by organism.** Organisms are

ordered along the x-axis by protein abundance (smallest to largest). See **Supplementary Table 1** for organism names and strains.

Supplementary Figure 3

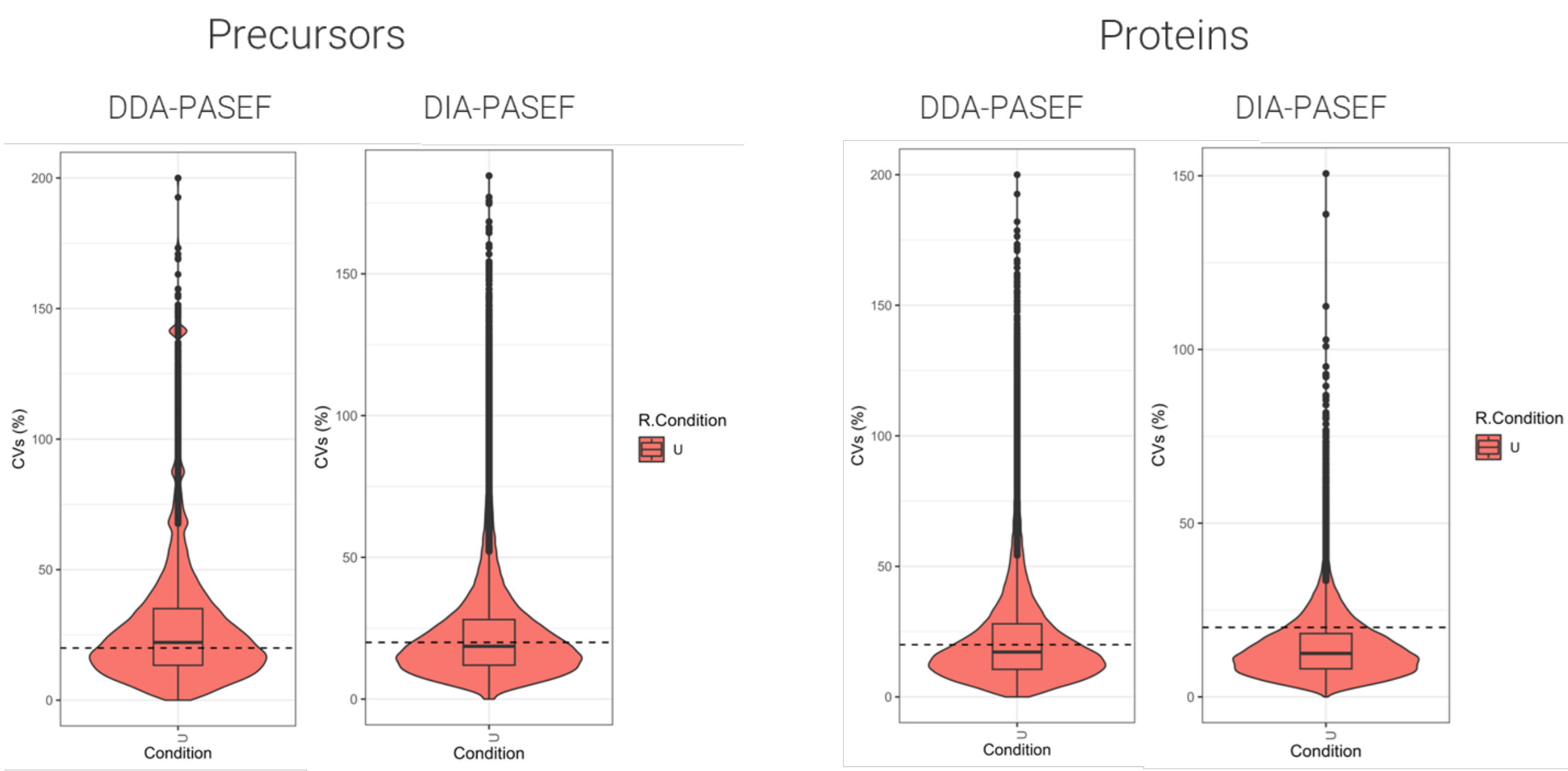

Supplementary Figure 3. Distribution of ddaPASEF and diaPASEF CV values. The distribution of CVs for ddaPASEF (DDA) and diaPASEF (DIA) methods were calculated at the precursor-level (left) and protein-level (right). The distribution of CV values for diaPASEF data sets indicates that the diaPASEF dataset has lower variances than those of the ddaPASEF dataset.

Supplementary Figure 4

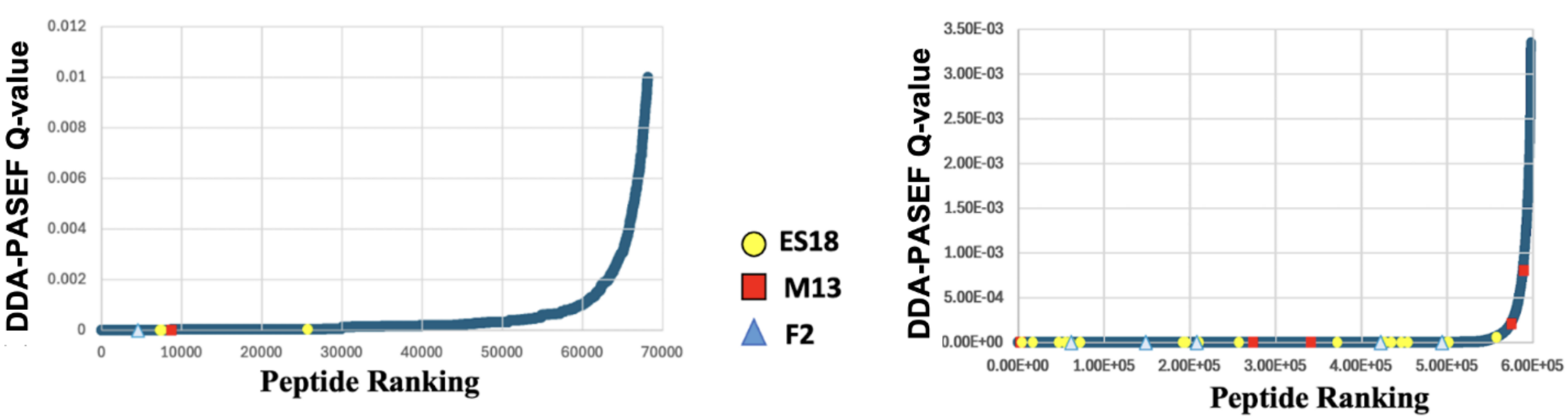

Supplementary Figure 4: Evidence for detection of five bacteriophages using Q-values. Q-values from peptide results for Spectromine (ddaPASEF) and Spectronaut (diaPASEF) were used for bacteriophages F2, ES18, and M13 to assess the confidence of their detection.
